# Bioassay-guided isolation of cytotoxic constituents from the flowers of *Aquilaria sinensis*

**DOI:** 10.1007/s13659-022-00334-3

**Published:** 2022-04-01

**Authors:** Jun Yang, Dong-Bao Hu, Meng-Yuan Xia, Ji-Feng Luo, Xing-Yu Li, Yue-Hu Wang

**Affiliations:** 1 Key Laboratory of Economic Plants and Biotechnology and Yunnan Key Laboratory for Wild Plant Resources, Kunming Institute of Botany, Chinese Academy of Sciences, Kunming, 650201 People’s Republic of China; 2 School of Chemical Biology and Environment, Yuxi Normal University, Yuxi, 653100 People’s Republic of China; 3 College of Science, Yunnan Agricultural University, Kunming, 650201 People’s Republic of China

**Keywords:** Thymelaeaceae, *Aquilaria sinensis*, Paclitaxel-resistant lung cancer cells, Cucurbitane-type triterpenoids, 2-(2-Phenylethyl)chromones

## Abstract

Bioassay-guided fractionation of the EtOH extract from the flowers of *Aquilaria sinensis* (Lour.) Spreng. (Thymelaeaceae) led to the isolation of a new cucurbitane-type triterpenoid, aquilarolide A (**1**), along with five known compounds (**2**–**6**). The structure of **1** was elucidated by extensive 1D and 2D nuclear magnetic resonance (NMR) experiments and mass spectrometry (MS) data and theoretical calculations of its electronic circular dichroism (ECD) spectra. Aquilarolide A, cucurbitacin E (**3**), cucurbitacin B (**4**), and 7-hydroxy-6-methoxy-2-[2-(4-methoxyphenyl)ethyl]-4*H*-1-benzopyran-4-one (**6**) showed significant cytotoxicity against human lung adenocarcinoma SPC-A-1, human lung squamous cell carcinoma NCI-H520, human lung adenocarcinoma A549, and paclitaxel-resistant A549 (A549/Taxol) cell lines. All four active compounds, with IC_50_ values ranging from 0.002 to 0.91 μM, had better inhibitory activities against A549/Taxol cells than paclitaxel (IC_50_ = 1.80 μM). Among them, cucurbitacin E (IC_50_ = 0.002 μM) is the most active. Further studies are needed to evaluate their in vivo antitumor activities and to clarify their mechanisms.

**Graphical Abstract:**

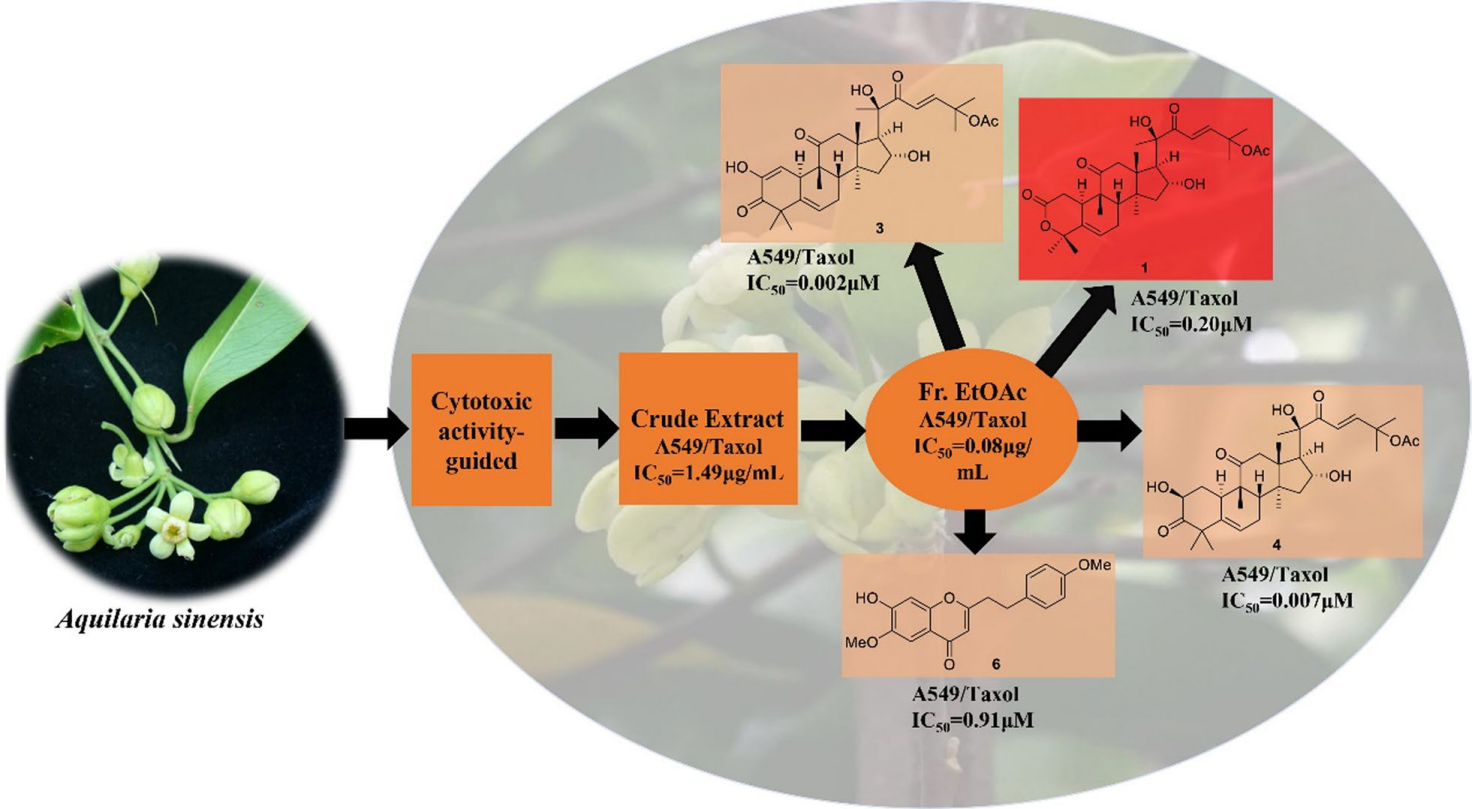

**Supplementary Information:**

The online version contains supplementary material available at 10.1007/s13659-022-00334-3.

## Introduction

Cancer is a major public health problem and one of the leading causes of mortality and morbidity worldwide [1]. Chemical drugs, such as paclitaxel, are useful to treat cancers. However, resistance to paclitaxel reduces the efficacy of chemotherapy and limits its clinical application [2]. Therefore, it is necessary to develop novel and effective therapeutic medicines or adjuvants for cancer. Natural plant resources are a rich source of anticancer agents. The discovery of new effective cancer drugs and understanding their underlying mechanism is one of the most studied topics among biologists and chemists.

*Aquilaria sinensis* (Lour.) Spreng. (Thymelaeaceae) is widely distributed in Hainan, Fujian, Yunnan, Guangdong, and Taiwan in China [3]. It has a particular economic interest because it is the principal source of agarwood (chen-xiang in Chinese), namely, the resinous heartwood of the plant. As a traditional Chinese medicine, agarwood has been widely investigated [4]. However, there have been only a few studies on the chemical constituents and bioactivities of *A. sinensis* flowers. The volatile constituents from flowers of *A. sinensis* have been analyzed by GC–MS [5]. Flavonoids and their glycosides are found in its flowers [6]. Benzophenone glycosides have been isolated from the flower buds of *A. sinensis* and two compounds, aquilasides B and C, displayed moderate cytotoxicity against SK-MEL cells with IC_50_ values of 17.0 and 12.0 μM, respectively [7].

**Fig. 1:**
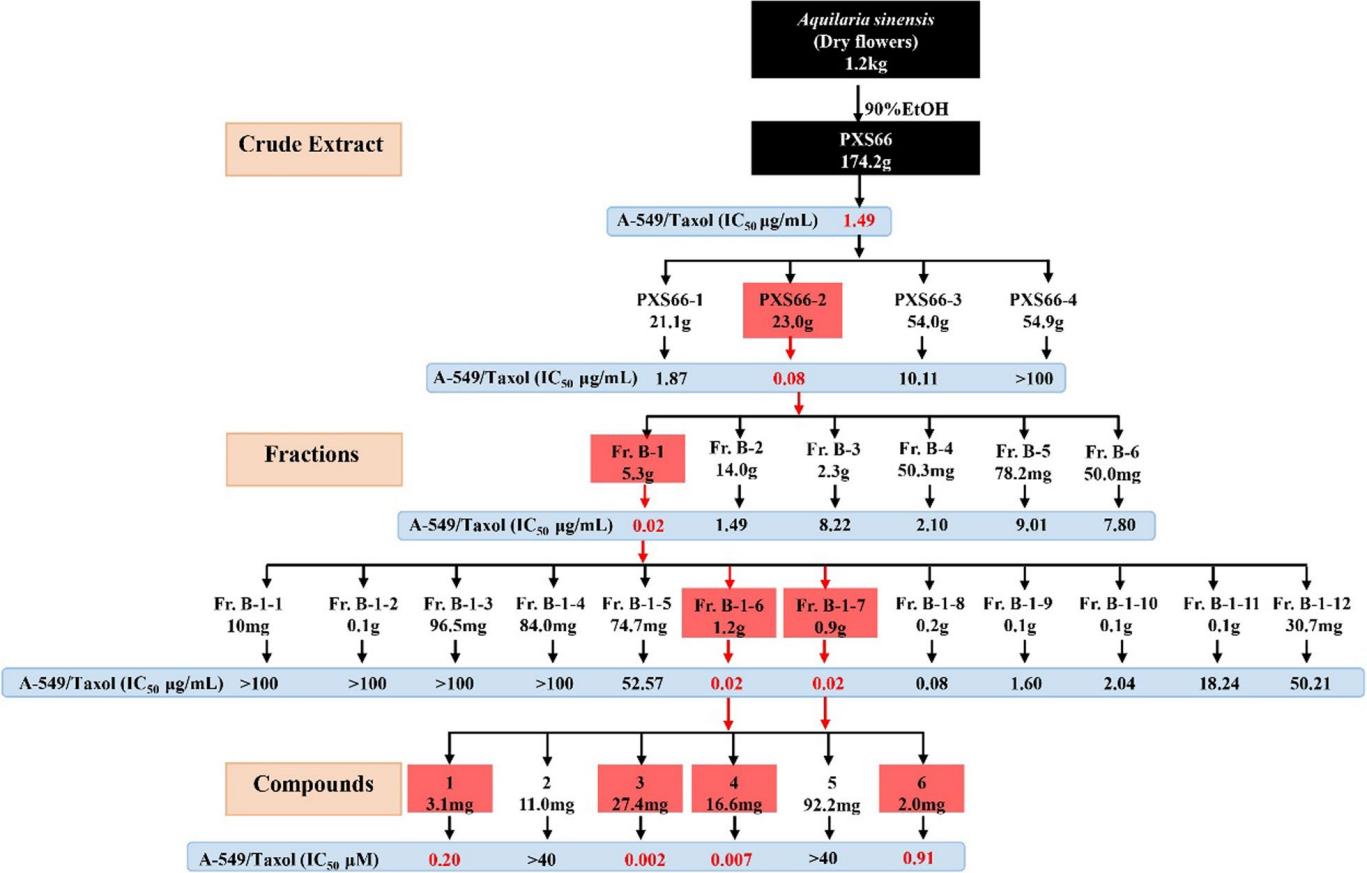
Schematic diagram showing cytotoxic compounds from *Aquilaria sinensis* flowers by bioassay-guided isolation

**Fig. 2:**
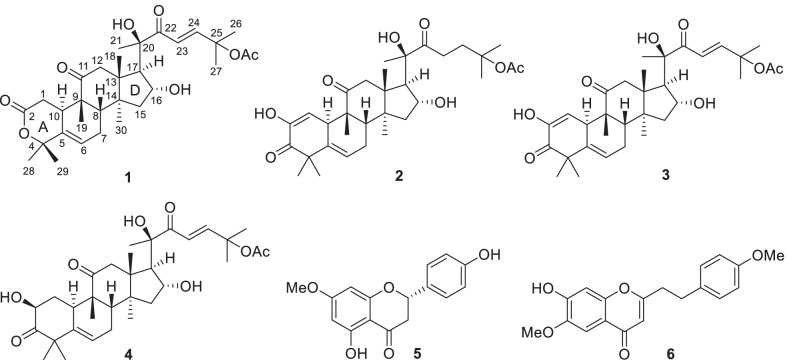
Chemical structures of isolates **1–6**

In our screening for anticancer extracts of plants, the extract (PXS65) of *A. sinensis* flowers was found to possess significant inhibitory activities against 16 cancer cell lines (Table 1), especially inhibiting lung cancer cell lines, such as human lung adenocarcinoma SPC-A-1 (IC_50_ = 0.11 μg/mL), human lung squamous cell carcinoma NCI-H520 (IC_50_ = 0.25 μg/mL), and human lung adenocarcinoma A549 (IC_50_ = 0.44 μg/mL) cells. Then, a bioassay-guided isolation of cytotoxic constituents against A549, NCI-H520, SPC-A-1, paclitaxel-resistant A549 (A549/Taxol), and human normal bronchial epithelial BEAS-2B cell lines was conducted (Fig. 1; Tables 2–5), which led to the isolation of four active compounds, including a new cucurbitane-type triterpenoid (**1**) (Fig. 2). The bioassay results and the structural elucidation of aquilarolide A (**1**) are reported.

**Table 1:** Cytotoxicity of the EtOH extract (PXS65) of *A. sinensis* after water extraction against 16 cancer cell lines and the normal human bronchial epithelial BEAS-2B cell line

No	Cell lines	IC_50_ (μg/mL)		
		PXS65	Cisplatin	Paclitaxel
1	SPC-A-1	0.11 ± 0.00	1.76 ± 0.40	< 0.007
2	NCI-H520	0.25 ± 0.02	6.69 ± 1.46	< 0.007
3	A549	0.44 ± 0.00	3.83 ± 0.79	< 0.007
4	HeLa	0.46 ± 0.01	1.14 ± 0.152	< 0.007
5	SH-SY5Y	0.48 ± 0.02	2.85 ± 0.272	0.008 ± 0.001
6	SK-OV-3	0.55 ± 0.03	3.67 ± 0.97	< 0.007
7	MT4	0.59 ± 0.074	0.16 ± 0.01	< 0.008
8	PC-3	0.84 ± 0.01	1.38 ± 0.23	< 0.007
9	SMMC-7721	1.36 ± 0.38	1.53 ± 0.11	0.15 ± 0.02
10	MDA-MB-231	1.39 ± 0.04	3.16 ± 0.97	< 0.007
11	NCI-H446	5.51 ± 0.41	3.78 ± 0.79	< 0.007
12	NCI-H460	12.24 ± 0.63	5.29 ± 0.48	< 0.007
13	SW480	21.94 ± 1.18	2.00 ± 0.52	< 0.007
14	MCF-7	33.29 ± 2.23	2.00 ± 0.93	< 0.007
15	HL-60	36.68 ± 1.20	1.97 ± 1.30	< 0.007
16	Caco2	> 40	3.24 ± 0.09	0.02 ± 0.02
17	BEAS-2B	28.73 ± 1.42	9.14 ± 1.41	3.66 ± 0.30

**Table 2:** Cytotoxicity of the EtOH extract (PXS66) and petroleum ether-soluble (PXS66-1), EtOAc-soluble (PXS66-2), *n*-BuOH-soluble (PXS66-3), and H_2_O-soluble (PSX66-4) fractions

Extracts/fractions	IC_50_ (μg/mL)				
	A-549	NCI-H520	SPC-A-1	A549/Taxol	BEAS-2B
PXS66	2.04 ± 0.01	0.72 ± 0.06	1.59 ± 0.15	1.49 ± 0.20	30.97 ± 0.76
PXS66-1	2.05 ± 0.08	1.39 ± 0.03	1.63 ± 0.05	1.87 ± 0.05	13.59 ± 0.57
PXS66-2	0.17 ± 0.02	0.08 ± 0.00	0.08 ± 0.00	0.08 ± 0.00	4.48 ± 0.16
PXS66-3	13.56 ± 0.32	11.06 ± 0.81	8.30 ± 0.15	10.11 ± 0.31	> 100
PXS66-4	> 100	> 100	> 100	> 100	> 100
Cisplatin	4.99 ± 0.08	3.12 ± 0.18	3.10 ± 0.13	4.29 ± 0.26	7.36 ± 0.56
Paclitaxel	< 0.007	< 0.007	< 0.007	0.54 ± 0.09	1.85 ± 0.19

## Results and discussion

### Bioactivity-guided fractionation and isolation

The 90% EtOH extract (PXS65) of *A. sinensis* flowers after water extraction was tested in vitro for its cytotoxicity against 16 cancer cell lines, including SPC-A-1, NCI-H520, A549, human cervical cancer HeLa, human neuroblastoma SH-SY5Y, human ovarian carcinoma SK-OV-3, human T-cell leukemia MT4, human prostate cancer PC-3, human hepatoma SMMC-7721, human breast cancer MDA-MB-231, human small cell lung cancer NCI-H446, human large cell lung carcinoma NCI-H460, human colon cancer SW-480, human breast cancer MCF-7, human leukemia HL-60, and human colon cancer Caco2 cell lines, as well as the normal BEAS-2B cell line by 3-(4,5-dimethylthiazol-2-yl)-5(3-carboxymethoxyphenyl)-2-(4-sulfopheny)-2*H*-tetrazolium (MTS) assay and their IC_50_ values (μg/mL) were determined (Table 1). PXS65 possessed pronounced cytotoxic activity against SPC-A-1, NCI-H520, A549, HeLa, SH-SY5Y, SK-OV-3, MT4, and PC-3 cells with IC_50_ values less than 1 μg/mL. Meanwhile, PXS65 exhibited weak inhibitory activity against SW480, MCF-7, HL-60, HL-60, Caco2, and BEAS-2B cells with IC_50_ values greater than 20 μg/mL. These data indicated that PXS65 had some selectivity for different cancerous cell lines and the normal BEAS-2B cell line. It had better inhibition against lung cancer cell lines than against other cancer cell lines (Table 1). Thus, the next bioactivity-guided separations were conducted according to the cytotoxicities of the fractions against lung cancer cell lines (A-549, NCI-H520, and SPC-A-1), as well as normal BEAS-2B cells and paclitaxel-resistant lung cancer A549/Taxol cells.

As shown in Table 2, the EtOAc-soluble fraction (PXS66-2) showed the most inhibitory activities against A-549 (IC_50_ = 0.17 μg/mL), NCI-H520 (IC_50_ = 0.08 μg/mL), SPC-A-1 (IC_50_ = 0.08 μg/mL), and A549/Taxol (IC_50_ = 0.08 μg/mL) cells. The inhibitory activity against A549/Taxol cells was better than that of paclitaxel (IC_50_ = 0.54 μg/mL), with lower toxicity (IC_50_ = 4.48 μg/mL) than that of paclitaxel (IC_50_ = 1.85 μg/mL) against normal BEAS-2B cells.

PXS66-2 was fractionated by silica gel column chromatography to yield six further fractions (B-1 to B-6), which were also submitted to a cytotoxicity assay (Table 3). Fr. B-1 showed observably higher inhibitory activities against these four lung cancer cell lines than other fractions with IC_50_ values less than or equal to 0.02 μg/mL. Fr. B-1 was separated by reverse-phase (RP) C_18_ silica gel column chromatography to yield 12 further fractions (B-1-1 to B-1-12), which were also submitted to a cytotoxicity assay (Table 4). Frs. B-1-6 and B-1-7 showed observably higher inhibitory activity against these four lung cancer cell lines than other fractions with IC_50_ values less than or equal to 0.02 μg/mL. Next, Frs. B-1-6 and B-1-7 were isolated and purified to yield six compounds (**1**–**6**) (Fig. 2).

**Table 3:** Cytotoxicity of the subfractions from the EtOAc-soluble fraction (PXS66-2) against the A549, NCI-H520, SPC-A-1, A549/Taxol, and BEAS-2B cell lines

Fractions	IC_50_ (μg/mL)				
	A549	NCI-H520	SPC-A-1	A549/Taxol	BEAS-2B
Fr. B-1	0.02 ± 0.00	0.01 ± 0.00	0.02 ± 0.00	0.02 ± 0.00	5.54 ± 0.32
Fr. B-2	2.16 ± 0.04	0.45 ± 0.03	1.76 ± 0.08	1.49 ± 0.11	49.83 ± 1.66
Fr. B-3	13.36 ± 1.02	6.44 ± 0.55	7.85 ± 0.07	8.22 ± 0.55	92.79 ± 0.92
Fr. B-4	5.84 ± 0.08	2.01 ± 0.14	9.57 ± 0.41	2.10 ± 0.06	> 100
Fr. B-5	35.10 ± 0.63	7.95 ± 0.07	29.72 ± 2.13	9.01 ± 0.21	> 100
Fr. B-6	12.97 ± 0.21	5.50 ± 0.50	11.33 ± 0.93	7.80 ± 0.19	> 100
Cisplatin	3.02 ± 0.59	3.75 ± 0.52	3.06 ± 1.07	3.41 ± 0.47	> 12
Paclitaxel	< 0.007	< 0.007	< 0.007	1.45 ± 0.12	2.04 ± 0.11

**Table 4:** Cytotoxicity of the subfractions from the active fraction (Fr. B-1) against the A549, NCI-H520, SPC-A-1, A549/Taxol, and BEAS-2B cell lines

Fractions	IC_50_ (μg/mL)				
	A549	NCI-H520	SPC-A-1	A549/Taxol	BEAS-2B
Fr. B-1–1	> 100	> 100	> 100	> 100	> 100
Fr. B-1–2	> 100	> 100	> 100	> 100	> 100
Fr. B-1–3	> 100	> 100	> 100	> 100	> 100
Fr. B-1–4	> 100	> 100	> 100	> 100	> 100
Fr. B-1–5	54.99 ± 2.81	41.33 ± 2.32	45.41 ± 0.91	52.57 ± 0.63	> 100
Fr. B-1–6	0.02 ± 0.00	0.01 ± 0.00	0.01 ± 0.00	0.02 ± 0.00	1.34 ± 0.05
Fr. B-1–7	0.01 ± 0.00	0.01 ± 0.00	0.01 ± 0.00	0.02 ± 0.00	1.42 ± 0.04
Fr. B-1–8	0.08 ± 0.00	0.02 ± 0.00	0.06 ± 0.00	0.08 ± 0.00	4.95 ± 0.19
Fr. B-1–9	0.35 ± 0.04	0.48 ± 0.03	0.35 ± 0.02	1.60 ± 0.06	13.20 ± 1.13
Fr. B-1–10	1.72 ± 0.10	1.20 ± 0.09	1.10 ± 0.08	2.04 ± 0.06	25.50 ± 0.84
Fr. B-1–11	11.04 ± 0.54	8.16 ± 0.46	8.27 ± 0.38	18.24 ± 0.61	26.46 ± 2.01
Fr. B-1–12	55.00 ± 1.83	36.70 ± 0.83	37.84 ± 2.03	50.21 ± 2.03	> 100
Cisplatin	2.28 ± 0.57	3.11 ± 0.22	2.49 ± 0.07	4.13 ± 0.86	8.86 ± 1.32
Paclitaxel	< 0.007	< 0.007	< 0.007	0.96 ± 0.05	1.94 ± 0.11

### Structural elucidation of isolates 1–6

In total, six secondary metabolites (Fig. 2), including a new metabolite (**1**), were isolated from the cytotoxically active fractions of *A. sinensis* flowers as a result of chromatographic separations. The chemical structure of the new compound was elucidated by 1D and 2D nuclear magnetic resonance (NMR) experiments as well as high-resolution electron ionization mass spectrometry (HRESIMS) and electronic circular dichroism (ECD) calculations.

Compound **1** was isolated as a white amorphous powder and exhibited a quasi-molecular ion peak at *m/z* 567.2937 [M + Na]^+^ in HRESIMS, suggesting a molecular formula of C_31_H_44_O_8_ (calcd. for C_31_H_44_NaO_8_, 567.2934) and 10 degrees of unsaturation. The ^1^H NMR spectrum (Table 1) of **1** indicated the presence of nine methyl groups at *δ*_H_ 0.98, 1.05, 1.33, 1.42, 1.51, 1.53, 1.54, 1.57, and 2.02 (methyl protons of an acetyl group) ppm, a *trans* double bond at *δ*_H_ 7.05 (d, *J* = 15.7 Hz) and 6.44 (d, *J* = 15.7 Hz) ppm, and a trisubstituted double bond at *δ*_H_ 5.75 (br s) ppm. The ^13^C NMR spectrum (Table 1) of **1** displayed 31 carbon signals indicating the presence of four carbonyl groups (*δ*_C_ 211.7, 202.4, 172.0, and 170.3), two double bonds (*δ*_C_ 152.1, 136.7, 120.6, and 120.2), nine methyl groups (*δ*_C_ 31.5, 30.6, 26.5, 25.9, 23.9, 22.0, 19.9, 18.5, and 18.4), four methylenes, four sp^3^ methines, and six quaternary carbons. These data showed a similar signal pattern with those of a lactone-type norcucurbitacin, neocucurbitacin E, except for the double bond at Δ^23^ in **1** [8].

**Fig. 3:**
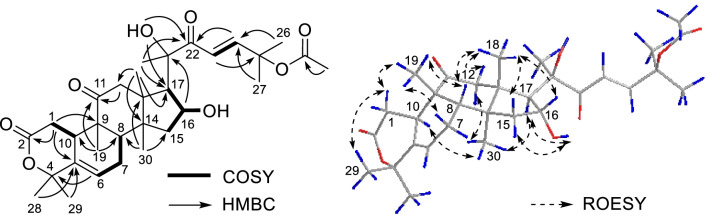
Key 2D NMR correlations of compound **1**

Based on the ^1^H–^1^H COSY correlations (Fig. 3), four connections, H_2_-1/H-10, H-6/H_2_-7/H-8, H_2_-15/H-16/H-17, and H-23/H-24, were deduced. The HMBC data revealed the lactone-type structure of ring A, similar to that of neocucurbitacin E [8], since H_2_-1 (*δ* 2.50 and 2.16) was correlated to the carbon atoms at *δ*_C_ = 172.0 (C-2), 136.7 (C-5), and 47.7 (C-9) ppm, as well as H_3_-28 and H_3_-29 to C-5 (Fig. 3) and H-6 to C-4. According to the HMBC correlations from H_3_-19 to C-8, C-10, and C-11, from H_3_-30 to C-8, C-13, and C-15, and from H_3_-18 to C-12, C-14, and C-17, rings B–D were deduced. Based on the HMBC correlations from H-16 to C-20, from H_3_-21 and 20-OH to C-17 and C-22, from H-23 to C-25, from H-24 to C-22, and from H_3_-26 and H_3_-27 to C-24, the side chain was confirmed and was located at C-17 of ring D. The acetyl group was located at C-25 (*δ*_C_ 79.4) by comparing the chemical shift of C-25 in 25-OH analogs (*δ*_C_ is approximately 71 ppm) and 25-OAc analogs (*δ*_C_ is approximately 79 ppm) [9]. Thus, the planar structure of **1** was determined as shown in Fig. 3.

**Fig. 4:**
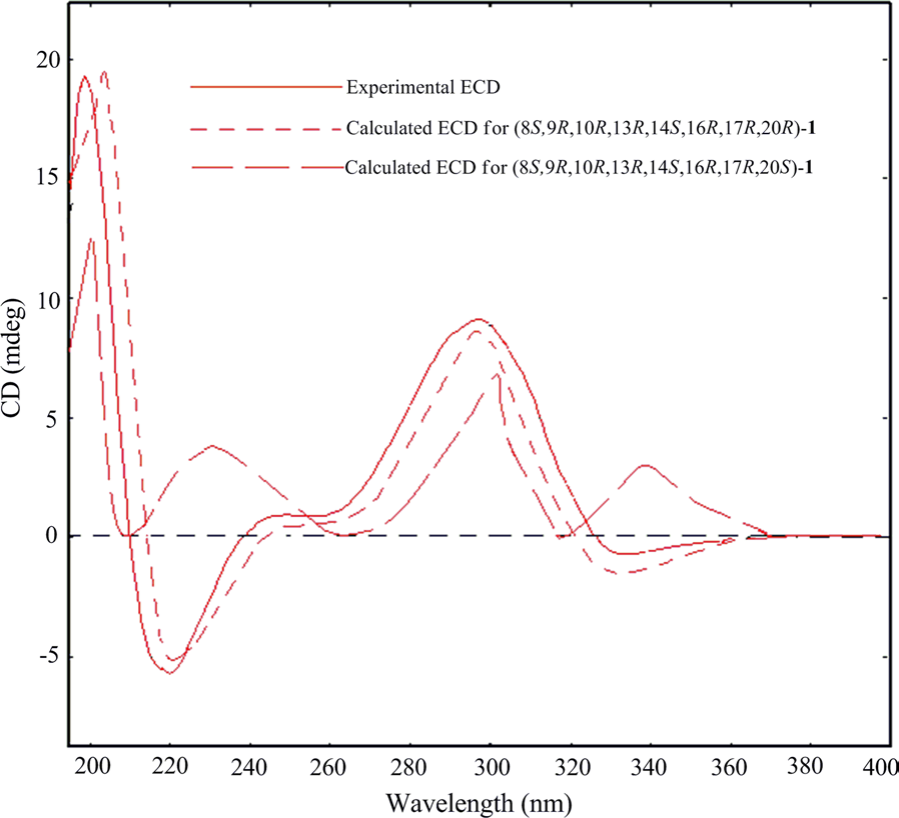
Experimental and calculated ECD spectra for compound **1**

The relative configuration of **1** was deduced by ROESY correlations (Fig. 3). Correlations of H-1*β*/H_3_-19, H-7*β*/H_3_-19, H-8/H_3_-18, H-8/H_3_-19, H-12*β*/H_3_-18, H-15*β*/H_3_-18, and H-16/H_3_-18 indicated that these protons should be *β*-oriented, while correlations of H-10/H_3_-30, H-12*α*/H_3_-30, H-17/H_3_-30, H-15*α*/16-OH, and H-17/16-OH showed that these protons should be *α*-oriented. The configuration of C-20 could not be determined by the ROESY spectrum. Accordingly, the ECD spectra of (*8S*,*9R*,*10R*,*13R*,*14S*,*16R*,*17R*,*20R*)-**1** and (*8S*,*9R*,*10R*,*13R*,*14S*,*16R*,*17R*,*20S*)-**1** were calculated (Fig. 4). The calculated ECD spectrum of (*8S*,*9R*,*10R*,*13R*,*14S*,*16R*,*17R*,*20R*)-**1** was similar to the experimental ECD spectrum of **1**. Thus, the absolute configuration of compound **1** was elucidated to be *8S*,*9R*,*10R*,*13R*,*14S*,*16R*,*17R*,*20R*, named aquilarolide A.

The known compounds were identified as 23,24-dihydrocucurbitacin E (**2**) [10], cucurbitacin E (**3**) [11], cucurbitacin B (**4**) [10], (−)-(2*S*)-5,4ʹ-dihydroxy-7-methoxyflavanone (**5**) [12], and 7-hydroxy-6-methoxy-2-[2-(4-methoxyphenyl)ethyl]-4*H*-1-benzopyran-4-one (**6**) [13] by comparison of the obtained spectroscopic data with those published in the literature.

### Cytotoxic results of isolates 1–6

Isolates **1**–**6** were evaluated for their cytotoxicities against SPC-A-1, NCI-H520, A549, A549/Taxol, and BEAS-2B cell lines (Table 5). Aquilarolide A (**1**), cucurbitacin E (**3**), cucurbitacin B (**4**), and 7-hydroxy-6-methoxy-2-[2-(4-methoxyphenyl)ethyl]-4*H*-1-benzopyran-4-one (**6**) displayed observable cytotoxicity against four tested cancer cell lines with IC_50_ values ranging from 0.001 to 1.84 μM and against the normal BEAS-2B cell line with IC_50_ values ranging from 3.46 to > 40 μM. All four active compounds, with activity strengths of **3** (IC_50_ = 0.002 μM) > **4** (IC_50_ = 0.007 μg/mL) > **1** (IC_50_ = 0.20 μM) > **6** (IC_50_ = 0.91 μM), had better inhibitory activities against A549/Taxol cells than paclitaxel (IC_50_ = 1.80 μM) (Table 5).

**Table 5:** Cytotoxicity of compounds isolated from the active fractions (Frs. B-1–6 and B-1–7) against the A549, NCI-H520, SPC-A-1, A549/Taxol, and BEAS-2B cell lines

Compounds	IC_50_ (μM)				
	A549	NCI-H520	SPC-A-1	A549/Taxol	BEAS-2B
**1**	0.35 ± 0.06	0.16 ± 0.01	0.56 ± 0.02	0.20 ± 0.01	17.93 ± 0.30
**2**	> 40	> 40	> 40	> 40	> 40
**3**	0.02 ± 0.00	0.001 ± 0.000	0.005 ± 0.000	0.002 ± 0.000	3.46 ± 0.13
**4**	0.03 ± 0.00	0.002 ± 0.000	0.016 ± 0.000	0.007 ± 0.001	14.42 ± 1.36
**5**	> 40	> 40	> 40	> 40	> 40
**6**	1.52 ± 0.06	1.84 ± 0.16	1.13 ± 0.03	0.91 ± 0.01	> 40
Cisplation	13.54 ± 0.64	11.95 ± 0.60	21.42 ± 0.35	14.95 ± 0.93	34.90 ± 1.16
Paclitaxel	< 0.008	< 0.008	< 0.008	1.80 ± 0.13	> 5

These active compounds belong to cucurbitane-type triterpenoids (**1**, **3**, and **4**) and a 2-(2-phenylethyl)chromone (**6**). This result agrees with those reported in the literature that cucurbitane-type triterpenoids were found to be the main constituents contributing to the cytotoxic activities in *A. sinensis* fruits and peels [14, 15].

Both cucurbotacin E (**3**) and 23,24-dihydrocucurbitacin E (**2**) have a four-ringed core structure in the cucurbitane skeleton, except for the side chain with an olefinic bond at C-23 in compound **3**. Cucurbitacin E showed significant cytotoxic activities against human lung cancer SPC-A-1, NCI-H520, A549, and A549/Taxol cell lines with IC_50_ values less than or equal to 0.02 μM. However, 23,24-dihydrocucurbitacin E (**2**) was inactive. This indicated that the side chain with the olefinic bond at C-23 seems to be key to the cytotoxic activity of this type of compound.

The inhibitory activities of cucurbitacins E (**3**) and B (**4**) were close to each other, with IC_50_ values less than or equal to 0.03 μM. However, the inhibitory activity of **1** was significantly weaker than that of compounds **3** and **4**. The difference between **1** and **3** and **4** is ring A. This indicates that the structure of ring A is also key to the cytotoxic activity of this type of compound. The cytotoxic potency of cucurbitacins in A549 cells was related to multivariate factors, among which the electrophilicity of molecules played a pivotal role, according to the multivariate structure–activity relationship (SAR) and quantitative structure–activity relationship modeling (QSAQ) analyses of cucurbitacin derivatives [16].

## Experimental section

### General experimental procedures

The reagents and instrumentation utilized for extraction, isolation, and structure characterization throughout this study are described in Additional file 1.

### Collection of plant samples

The flowers of *Aquilaria sinensis* were collected from Menghai County, Yunnan Province, China, in 2019. A voucher specimen (No. KIB001-003) was identified by Ms. Jun Yang at Kunming Institute of Botany, Chinese Academy of Sciences.

### Preparation of extractions and fractions and isolation of compounds

Air-dried, powdered flowers (50.0 g) of *A. sinensis* were extracted under ultrasound with H_2_O (500 mL × 3) at 60 ℃ for 30 min. The remaining residue was further extracted with 90% EtOH (500 mL × 3) at 60 ℃ for 30 min and the solvent was removed to yield crude extract PXS65 (2.4 g).

Air-dried, powdered flowers (1.2 kg) of *A. sinensis* were extracted under ultrasound with 90% EtOH (2 L × 4) at 60 ℃ for 30 min and the solvent was removed to yield crude extract PXS66 (174.2 g). PXS66 was suspended in water (500 mL) and then partitioned in turn with petroleum ether (500 mL × 4), EtOAc (500 mL × 4), and *n*-BuOH (500 mL × 4) to yield three fractions, PXS66-1 (21.1 g), PXS66-2 (23.0 g), and PXS66-3 (54.0 g), respectively. The solvent in the remaining water phase was removed to yield PXS66-4 (54.9 g).

PXS66-2 (23.0 g) was subjected to column chromatography (silica gel; CH_2_Cl_2_/MeOH, 1:0 → 0:1, v/v) to yield six further fractions (B-1–B-6). Fr. B-1 was separated on an RP C_18_ silica gel column eluted with MeOH/H_2_O (5% → 100%) to yield twelve further fractions (B-1-1–B-1-12). The 50% MeOH-eluted portion (Fr. B-1–6) was purified by column chromatography (silica gel; petroleum ether/EtOAc, 5:1 → 0:1, v/v) to yield six further fractions (B-1-6-1–B-1-6-6). Fr. B-1-6-1 was recrystallized from MeOH to yield **5** (92.2 mg). Fr. B-1-6-3 was purified by Sephadex LH-20 column chromatography (MeOH) and recrystallized from MeOH to yield **2** (11.0 mg). Fr. B-1-6-4 was recrystallized from MeOH to yield **3** (8.9 mg), and the remaining mother liquor was subjected to Sephadex LH-20 column chromatography (MeOH) to yield four further fractions (B-1-6-4-1–B-1-6-4-4). Fr. B-1-6-4-1 (33.5 mg) was purified by semipreparative high-performance liquid chromatography (HPLC) (Welch Ultimate AQ-C_18_, 7.8 × 250 mm, MeOH/H_2_O, 20:70, *v* = 2 mL/min) to yield **1** (3.1 mg, *t*_R_ = 24.543 min) and **4** (16.6 mg, *t*_R_ = 28.464 min). Fr. B-1–6-4–2 (72.6 mg) was purified by semipreparative HPLC (Welch Ultimate AQ-C_18_, 7.8 × 250 mm, MeOH/H_2_O, 15:85, *v* = 2 mL/min) to yield **6** (2.0 mg, *t*_R_ = 27.467 min). The 60% MeOH-eluted portion (Fr. B-1–7) was purified by column chromatography (silica gel; petroleum ether/EtOAc, 5:1 → 0:1, v/v) to yield six further fractions (B-1-7-1–B-1-7-6). Fr. B-1-7-5 and Fr. B-1–7-6 were recrystallized from MeOH to yield **3** (18.5 mg).

Aquilarolide A (**1**). White amorphous powder; [*α*]_D_^24^ − 7.1 (*c* 0.10, MeOH); ECD (*c* 0.056, MeOH) *λ*_max_ (Δ*ε*) 334 (− 0.22), 298 (+ 2.68), 219 (− 1.67), 199 (+ 5.67) nm; UV (MeOH) *λ*_max_ 282 (2.70), 229 (3.84) nm; ^1^H and ^13^C NMR data, see Table 6; ESI–MS *m/z* 567 [M + Na]^+^; HRESIMS *m/z* 567.2937 [M + Na]^+^ (calcd. for C_31_H_44_NaO_8_, 567.2934) (Additional file 1).

**Table 6:** ^1^H and ^13^C NMR data of compound **1** in CDCl_3_ (*δ* in ppm, *J* in Hz)

No	*δ*_H_ (500 MHz)	*δ*_C_ (126 MHz)
1*α* 1*β*	2.50, 1H, dd (16.2, 3.8) 2.16, 1H, dd (16.2, 13.9)	30.3
2		172.0
4		83.9
5		136.7
6	5.75, 1H, br s	120.6
7*α*	2.05, 1H, m	23.7
7*β*	2.40, 1H, m	
8	2.02, 1H, m	42.1
9		47.7
10	2.76, 1H, m	33.2
11		211.7
12*α*	3.11, d (14.9)	48.7
12*β*	2.68, d (14.9)	
13		50.4
14		48.0
15*α*	1.46, 1H, m	45.5
15*β*	1.88, 1H, dd (13.2, 9.8)	
16	4.36, 1H, m	71.3
17	2.45, 1H, br d (7.1)	58.2
18	0.98, 3H, s	19.9
19	1.05, 3H, s	18.5
20		78.1
21	1.42, 3H, s	23.9
22		202.4
23	6.44, 1H, d (15.7)	120.2
24	7.05, 1H, d (15.7)	152.1
25		79.4
26	1.54, 3H, s	26.5
27	1.57, 3H, overlapped	25.9
28	1.53, 3H, s	30.6
29	1.51, 3H, s	31.5
30	1.33, 3H, s	18.4
16-OH	1.75, 1H, d (6.7)	
20-OH	4.28, 1H, s	
25-OAc		170.3
	2.02, 3H, s	22.0

### MTS assay for cytotoxicity

The cytotoxicity activities were evaluated by MTS assay as previously described [17].

### Computational methods

The absolute configuration of the new compound was determined by time-dependent density functional theory (TDDFT) calculations of ECD spectra according to our previously published paper [18].

## Conclusion

In this study, bioassay-guided fractionation and purification were used to isolate the cytotoxic compounds of the extract from *A. sinensis* flowers. First, the crude extract showed significant inhibitory activities against 16 cancer cell lines with the most significant activities against the lung cancer SPC-A-1, NCI-H520, and A549 cell lines. Second, all fractions, subfractions, and pure compounds were screened for their cytotoxic activity against lung cancer SPC-A-1, NCI-H520, A549, and A549/Taxol cell lines and normal human bronchial epithelial BEAS-2B cells. From the active fraction, six compounds, including a new cucurbitane-type triterpenoid, aquilarolide A (**1**), five known compounds, namely, 23,24-dihydrocucurbitacin E (**2**), cucurbitacin E (**3**), cucurbitacin B (**4**), (−)-(2*S*)-5,4ʹ-dihydroxy-7-methoxyflavanone (**5**), and 7-hydroxy-6-methoxy-2-[2-(4-methoxyphenyl)ethyl]-4*H*-1-benzopyran-4-one (**6**), were identified. Compounds **1**, **3**, **4**, and **6** showed significant cytotoxicity activities against these four human lung cancer cell lines. All four active compounds, with activity strengths of **3** > **4** > **1** > **6**, had better inhibitory activities against A549/Taxol cells than paclitaxel. Further studies are needed to evaluate in vivo antitumor activities and clarify the mechanisms of these active compounds.

## Supplementary Information

**Additional file 1.** General experimental procedures, computational methods for the ECD of compound 1, and NMR, HRESIMS, and ECD spectra of compound **1**.
