# Supplementary material for: Bioassay-guided isolation of cytotoxic constituents from the flowers of Aquilaria sinensis
Source: Nat Prod Bioprospect. 2022 Apr 1;12(1):11. doi: 10.1007/s13659-022-00334-3 (PMC8975978; doi:10.1007/s13659-022-00334-3)
Supplement: Supplementary file 1 — Additional file 1. General experimental procedures, computational methods for the ECD of compound 1, and NMR, HRESIMS, and ECD spectra of compound 1. [file 13659_2022_334_MOESM1_ESM.pdf]

Supplementary Material for

Bioassay-guided isolation of cytotoxic constituents from the flowers of *Aquilaria sinensis*

Jun Yang[1†], Dong-Bao Hu[2†], Meng-Yuan Xia[1], Ji-Feng Luo[1], Xing-Yu Li[3] and Yue-Hu Wang[1*]

Contents

Title	Page
**General Experimental Procedures**	2
**Computational methods for ECD of compound 1**	3
**Fig. S1** ^{1}H NMR spectrum of **1** ($CDCl_3$, 500 MHz)	17
**Fig. S2** ^{13}C NMR spectrum of **1** ($CDCl_3$, 126 MHz)	18
**Fig. S3** HSQC spectrum of **1**	19
**Fig. S4** ^{1}H–^{1}H COSY spectrum of **1**	20
**Fig. S5** HMBC spectrum of **1**	21
**Fig. S6** ROESY spectrum of **1**	22
**Fig. S7** HRESIMS spectrum of **1**	23
**Fig. S8** ECD spectrum of **1**	24

[*]Correspondence: wangyuehu@mail.kib.ac.cn
[†]Jun Yang and Dong-Bao Hu contributed equally to this work
[1]Key Laboratory of Economic Plants and Biotechnology and Yunnan Key Laboratory for Wild Plant Resources, Chinese Academy of Sciences, Kunming 650201, People's Republic of China. [2]School of Chemical Biology and Environment, Yuxi Normal University, Yuxi 653100, People's Republic of China. [3]College of Science, Yunnan Agricultural University, Kunming 650201, People's Republic of China.

General experimental procedures

Optical rotations were recorded using a JASCO P-1020 Polarimeter (Jasco Corp., Tokyo, Japan). Ultraviolet (UV) spectra were taken on a Shimadzu UV-2401 PC spectrophotometer (Shimadzu, Kyoto, Japan). Electronic circular dichroism (ECD) spectra were recorded on a Chirascan CD spectrometer (Applied Photophysics Ltd., Leatherhead, UK). ^{1}H and ^{13}C nuclear magnetic resonance (NMR) spectra were collected on a Bruker AM-400, a Bruker DRX-500, a Bruker Avance III-600, and a Bruker Accend™ 800 MHz spectrometers (Bruker Corp., Karlsruhe, Germany) with tetramethylsilane (TMS) as an internal standard. Electrospray ionization mass spectrometry (ESIMS) and high-resolution electrospray ionization mass spectrometry (HRESIMS) analyses were performed on an API QSTAR Pulsar 1 spectrometer (Applied Biosystems/MDS Sciex, Foster City, CA, USA). Silica gel G (80–100 and 300–400 mesh, Qingdao Meigao Chemical Co., Ltd., Qingdao, China), C_{18} silica gel (40–75 µm, Fuji Silysia Chemical Ltd., Aichi, Japan), and Sephadex LH-20 (GE Healthcare Bio-Sciences AB, Uppsala, Sweden) were used for column chromatography, and silica gel GF_{254} (Qingdao Meigao Chemical Co., Ltd.) was used for preparative thin layer chromatography (TLC) as precoated plates. TLC spots were visualized under UV light at 254 nm and by dipping into 5% H_2SO_4 in alcohol followed by heating. Semipreparative high-performance liquid chromatography (HPLC) was performed on an Agilent 1200 series pump (Agilent Technologies, Santa Clara, USA) equipped with a diode array detector and a Welch Ultimate AQ-C_{18} column (5.0 µm, ϕ 7.8 × 250 mm).

Computational methods for ECD of compound 1

The theoretical calculations of compound **1** were performed using Gaussian 09 [1] and figured using GaussView 5.0 [2]. Conformation search using molecular mechanics calculations was performed in Discovery Studio 3.5 Client with MMFF force field with 20 kcal mol^{-1} upper energy limit [3].

The optimized conformation geometries and thermodynamic parameters of all selected conformations were provided. The predominant conformers were optimized at B3LYP/6-31G(d,p) level. The theoretical calculation of ECD was performed using time-dependent density functional theory (TDDFT) at B3LYP/6-31G(d,p) level in MeOH with PCM model [4]. The ECD spectra of compound **1** were obtained by weighing the Boltzmann distribution rate of each geometric conformation [5].

The ECD spectra were simulated by overlapping Gaussian functions for each transition according to:

$$\Delta\varepsilon(E) = \frac{1}{2.297 \times 10^{-39}} \times \frac{1}{\sqrt{2\pi\sigma}} \sum_{i}^{A} \Delta E_i R_i e^{-[(E-E_i)/(2\sigma)]^2} \quad (1)$$

The σ represented the width of the band at 1/*e* height, and ΔE_i and R_i were the excitation energies and rotational strengths for transition *i*, respectively. R_{vel} had been used in this work.

[1] Gaussian 09, Revision C.01,M. J. Frisch, G. W. Trucks, H. B. Schlegel, G. E. Scuseria, M. A. Robb, J. R. Cheeseman, G. Scalmani, V. Barone, B. Mennucci, G. A. Petersson, H. Nakatsuji, M. Caricato, X. Li, H. P. Hratchian, A. F. Izmaylov, J. Bloino, G. Zheng, J. L. Sonnenberg, M. Hada, M. Ehara, K. Toyota, R. Fukuda, J. Hasegawa, M. Ishida, T. Nakajima, Y. Honda, O. Kitao, H. Nakai, T. Vreven, J. A. Montgomery, Jr., J. E. Peralta, F. Ogliaro, M. Bearpark, J. J. Heyd, E. Brothers, K. N. Kudin, V. N. Staroverov, T. Keith, R. Kobayashi, J. Normand, K. Raghavachari, A. Rendell, J. C. Burant, S. S. Iyengar, J. Tomasi, M. Cossi, N. Rega, J. M. Millam, M. Klene, J. E. Knox, J. B. Cross, V. Bakken, C. Adamo, J. Jaramillo, R. Gomperts, R. E. Stratmann, O. Yazyev, A. J. Austin, R. Cammi, C. Pomelli, J. W. Ochterski, R. L. Martin, K. Morokuma, V. G. Zakrzewski, G. A. Voth, P. Salvador, J. J. Dannenberg, S. Dapprich, A. D. Daniels, O. Farkas, J. B. Foresman, J. V. Ortiz, J. Cioslowski, and D. J. Fox, Gaussian, Inc., Wallingford CT, 2010.

[2] GaussView, Version 5, Dennington, R.; Keith, T.; Millam, J. *Semichem Inc.*, Shawnee Mission, KS, **2009**.

[3] Smith, S. G.; Goodman, J. M. *J. Am. Chem. Soc.* **2010**, *132*, 12946−12959.

[4] (a) Miertus, S.; Scrocc, E.; Tomasi, J. *J. Chem. Phys.* **1981**, *55*, 117. (b) Miertus, S.; Tomasi, J. *J. Chem. Phys.* **1982**, *65*, 239. (c) Cossi, M.; Barone, V.; Cammi, R.; Tomasi, J. *Chem. Phys. Lett.* **1996**, *255*, 327.

[5] Tähtinen, P.; Bagno, A.; Klika, K. D.; Pihlaja, K. *J. Am. Chem. Soc.* **2003**, *125*, 4609−4618.

Calculated (8*S*,9*R*,10*R*,13*R*,14*S*,16*R*,17*R*,20*R*)-1

Center Number	Atomic Number	Atomic Type	Coordinates (Angstroms) X	Y	Z
1	1	0	4.248730	0.540505	-2.533318
2	6	0	4.611435	0.823913	-1.544215
3	1	0	5.595850	0.359009	-1.414272
4	6	0	3.649706	0.383920	-0.418709
5	1	0	2.732669	0.968756	-0.572022
6	6	0	4.235363	0.810562	0.912294
7	6	0	5.007426	2.137991	0.929340
8	6	0	4.770633	2.328655	-1.526007
9	8	0	4.818406	3.026347	-2.509985
10	8	0	4.795846	2.908799	-0.295738
11	6	0	3.264041	-1.143871	-0.478294
12	6	0	3.378246	-1.241464	2.094362
13	1	0	4.117738	-2.029385	2.303052
14	1	0	2.722361	-1.238314	2.975561
15	6	0	4.103719	0.072015	2.018675
16	1	0	4.560602	0.408308	2.949070
17	6	0	6.522015	1.915837	1.079496
18	1	0	6.915328	1.278433	0.281369
19	1	0	6.742607	1.429848	2.035174
20	1	0	7.044687	2.877132	1.042579
21	6	0	4.490018	3.086022	2.018577
22	1	0	4.656607	2.667282	3.014922
23	1	0	3.418447	3.263888	1.890133
24	1	0	5.012865	4.044955	1.950943
25	6	0	2.348278	-1.280720	-1.727501
26	6	0	0.834692	-1.080316	-1.585097
27	1	0	0.396535	-1.422609	-2.525092
28	1	0	0.622163	-0.006344	-1.508325
29	6	0	1.042030	-1.296216	0.932081
30	8	0	2.828952	-1.505748	-2.824044
31	6	0	-2.348584	-2.319816	-0.736122
32	6	0	-3.709339	-1.875031	-0.124003
33	6	0	-4.114097	-0.454402	-0.177195
34	1	0	-3.409219	0.279396	-0.548175
35	6	0	-5.338167	-0.083298	0.219271
36	1	0	-6.013415	-0.855501	0.584998
37	8	0	-4.441903	-2.752046	0.327391
38	6	0	-5.907338	1.310967	0.232669
39	6	0	-6.225460	1.703094	1.687415

40	1	0	-5.309465	1.698791	2.286376
41	1	0	-6.922320	0.978436	2.121940
42	1	0	-6.678643	2.694940	1.724167
43	6	0	-7.155498	1.358734	-0.666764
44	1	0	-6.886314	1.114821	-1.699357
45	1	0	-7.608530	2.350808	-0.636811
46	1	0	-7.889493	0.622876	-0.321584
47	8	0	-4.856446	2.169555	-0.309603
48	6	0	-5.027078	3.508258	-0.433142
49	8	0	-6.027421	4.116767	-0.122391
50	6	0	-3.781620	4.135663	-1.019045
51	1	0	-3.927261	5.212677	-1.107145
52	1	0	-3.571241	3.707542	-2.004688
53	1	0	-2.917139	3.927537	-0.380167
54	6	0	2.556385	-1.629893	0.841936
55	1	0	2.598739	-2.722679	0.801626
56	6	0	4.528712	-2.002364	-0.693112
57	1	0	4.286240	-3.067389	-0.607027
58	1	0	5.291729	-1.768178	0.056119
59	1	0	4.947878	-1.848158	-1.688166
60	6	0	0.307242	-1.849084	-0.359225
61	6	0	-1.185879	-1.649776	0.058034
62	1	0	-1.382886	-0.572925	0.037298
63	6	0	-1.203564	-2.076765	1.551060
64	1	0	-1.611181	-3.090623	1.628224
65	6	0	0.270862	-2.044059	2.049199
66	1	0	0.653753	-3.060805	2.191980
67	1	0	0.329704	-1.535350	3.017334
68	6	0	0.597809	-3.351622	-0.622380
69	1	0	0.344757	-3.994843	0.222560
70	1	0	1.646392	-3.528926	-0.881121
71	1	0	-0.001619	-3.703151	-1.464212
72	6	0	0.780998	0.215290	1.190933
73	1	0	-0.236785	0.389463	1.546253
74	1	0	0.939220	0.856166	0.320635
75	1	0	1.451984	0.572344	1.977643
76	8	0	-2.064768	-1.162699	2.243292
77	1	0	-2.328941	-1.574200	3.080377
78	6	0	-2.369392	-1.945536	-2.233728
79	1	0	-3.291489	-2.312697	-2.696955
80	1	0	-1.532830	-2.427561	-2.744055
81	1	0	-2.303225	-0.865216	-2.398483
82	8	0	-2.290676	-3.730757	-0.622448
83	1	0	-3.153085	-3.980931	-0.225960

```
 -------------------------------------------------------------------
Excitation energies and oscillator strengths:
 Excited State   1:      Singlet-A      3.9498 eV  313.90 nm  f=0.0018  <S**2>=0.000
     142 ->148        -0.22623
     143 ->148         0.54328
     144 ->148         0.13094
     147 ->148        -0.33487
 This state for optimization and/or second-order correction.
 Total Energy, E(TD-HF/TD-KS) =  -1809.48291866
 Copying the excited state density for this state as the 1-particle RhoCI density.

 Excited State   2:      Singlet-A      4.1562 eV  298.31 nm  f=0.0008  <S**2>=0.000
     143 ->149         0.10676
     146 ->149         0.20794
     147 ->149         0.65388

 Excited State   3:      Singlet-A      4.3864 eV  282.65 nm  f=0.0040  <S**2>=0.000
     142 ->148        -0.14506
     143 ->148         0.27101
     144 ->148         0.11210
     147 ->148         0.61732

 Excited State   4:      Singlet-A      4.6059 eV  269.19 nm  f=0.0251  <S**2>=0.000
     143 ->148        -0.16046
     144 ->148         0.62644
     146 ->148        -0.26456

 Excited State   5:      Singlet-A      4.6573 eV  266.21 nm  f=0.0007  <S**2>=0.000
     144 ->148         0.24604
     146 ->148         0.65116

 Excited State   6:      Singlet-A      4.8385 eV  256.25 nm  f=0.0537  <S**2>=0.000
     140 ->148        -0.16344
     142 ->148         0.55425
     143 ->148         0.26758
     145 ->148        -0.29097

 Excited State   7:      Singlet-A      4.9057 eV  252.73 nm  f=0.0051  <S**2>=0.000
     142 ->148         0.28371
     145 ->148         0.63872

 Excited State   8:      Singlet-A      5.0796 eV  244.08 nm  f=0.0008  <S**2>=0.000
     141 ->148         0.70238
```

Excited State 9: Singlet-A 5.4043 eV 229.42 nm f=0.0007 <S**2>=0.000
146 ->149 0.66734
147 ->149 -0.22421

Excited State 10: Singlet-A 5.5173 eV 224.72 nm f=0.3729 <S**2>=0.000
136 ->148 0.18007
138 ->148 0.13617
140 ->148 0.62398
142 ->148 0.16602

Excited State 11: Singlet-A 5.5476 eV 223.49 nm f=0.0067 <S**2>=0.000
145 ->149 -0.25874
145 ->152 0.63072
147 ->152 0.11774

Excited State 12: Singlet-A 5.6859 eV 218.05 nm f=0.0009 <S**2>=0.000
141 ->150 0.69291

Excited State 13: Singlet-A 5.7115 eV 217.08 nm f=0.0098 <S**2>=0.000
133 ->148 0.14224
138 ->148 0.59152
139 ->148 -0.26748
140 ->148 -0.11929
143 ->148 -0.10558

Excited State 14: Singlet-A 5.7199 eV 216.76 nm f=0.0017 <S**2>=0.000
138 ->148 0.23467
139 ->148 0.64415
145 ->149 -0.10736

Excited State 15: Singlet-A 5.7268 eV 216.50 nm f=0.0086 <S**2>=0.000
145 ->149 0.63932
145 ->152 0.24978

Excited State 16: Singlet-A 5.9206 eV 209.41 nm f=0.0026 <S**2>=0.000
146 ->151 0.15938
147 ->151 0.68042

Excited State 17: Singlet-A 6.0007 eV 206.62 nm f=0.0241 <S**2>=0.000
135 ->148 -0.12325
136 ->148 0.43947
143 ->149 -0.40381
144 ->149 0.28803

Excited State 18: Singlet-A 6.0055 eV 206.45 nm f=0.0189 <S**2>=0.000
135 ->148 -0.13789
136 ->148 0.46750
140 ->148 -0.11393
143 ->149 0.35874
144 ->149 -0.29051

Excited State 19: Singlet-A 6.0558 eV 204.74 nm f=0.0153 <S**2>=0.000
133 ->148 -0.22668
134 ->148 -0.18425
135 ->148 0.42802
136 ->148 0.16460
137 ->148 -0.39365
144 ->149 -0.13875

Excited State 20: Singlet-A 6.0698 eV 204.26 nm f=0.0022 <S**2>=0.000
137 ->148 -0.11095
142 ->149 0.10625
143 ->149 0.36239
144 ->149 0.55382

Excited State 21: Singlet-A 6.1457 eV 201.74 nm f=0.0055 <S**2>=0.000
132 ->148 0.15103
135 ->148 0.36776
137 ->148 0.40514
142 ->149 0.36973

Excited State 22: Singlet-A 6.1583 eV 201.33 nm f=0.0063 <S**2>=0.000
135 ->148 -0.25672
137 ->148 -0.26525
142 ->149 0.55402
143 ->149 -0.11624

Excited State 23: Singlet-A 6.1713 eV 200.91 nm f=0.0018 <S**2>=0.000
129 ->148 0.11940
132 ->148 0.25049
133 ->148 0.54030
134 ->148 -0.14244
137 ->148 -0.23725
138 ->148 -0.19463

Excited State 24: Singlet-A 6.2657 eV 197.88 nm f=0.0000 <S**2>=0.000
147 ->150 0.70567

Excited State 25: Singlet-A 6.3217 eV 196.12 nm f=0.0003 <S**2>=0.000
145 ->152 -0.10032
146 ->152 -0.18723
147 ->152 0.66738

Excited State 26: Singlet-A 6.3446 eV 195.42 nm f=0.0003 <S**2>=0.000
145 ->151 0.69915

Excited State 27: Singlet-A 6.4894 eV 191.06 nm f=0.0036 <S**2>=0.000
132 ->148 -0.27978
133 ->148 0.21084
134 ->148 0.53139
135 ->148 0.22799
137 ->148 -0.14677

Excited State 28: Singlet-A 6.5057 eV 190.58 nm f=0.0046 <S**2>=0.000
146 ->152 0.67352
147 ->152 0.17755

Excited State 29: Singlet-A 6.5402 eV 189.57 nm f=0.0037 <S**2>=0.000
138 ->149 0.22843
139 ->149 0.42249
140 ->149 0.39882
141 ->149 -0.26575
143 ->149 0.11975

Excited State 30: Singlet-A 6.5524 eV 189.22 nm f=0.0027 <S**2>=0.000
132 ->148 0.12086
137 ->149 0.12332
138 ->149 -0.13500
139 ->149 -0.33917
140 ->149 0.50809
142 ->149 0.13002
146 ->150 0.14147
146 ->151 0.11449

Calculated (8*S*,9*R*,10*R*,13*R*,14*S*,16*R*,17*R*,20*S*)-1

Center Number	Atomic Number	Atomic Type	Coordinates (Angstroms) X	Y	Z
1	1	0	-2.250051	1.847948	1.689768
2	6	0	-3.043444	1.399667	1.090580
3	1	0	-3.890933	1.211176	1.759301
4	6	0	-2.590357	0.097172	0.396888
5	1	0	-1.822212	0.405746	-0.323240
6	6	0	-3.747787	-0.467915	-0.401983
7	6	0	-4.731583	0.546990	-1.001808
8	6	0	-3.447901	2.397803	0.030255
9	8	0	-3.118236	3.562066	0.016951
10	8	0	-4.174936	1.903918	-1.002542
11	6	0	-1.937595	-0.949950	1.371595
12	6	0	-2.997191	-2.841872	-0.007442
13	1	0	-3.571086	-3.427883	0.726245
14	1	0	-2.747881	-3.559921	-0.800672
15	6	0	-3.907130	-1.784728	-0.572243
16	1	0	-4.758570	-2.157591	-1.140585
17	6	0	-6.060965	0.587028	-0.228928
18	1	0	-5.909257	0.812057	0.831055
19	1	0	-6.564163	-0.382831	-0.295322
20	1	0	-6.715773	1.354380	-0.654547
21	6	0	-4.986447	0.290784	-2.492303
22	1	0	-5.482074	-0.671745	-2.645763
23	1	0	-4.042952	0.290234	-3.045187
24	1	0	-5.627315	1.080096	-2.896656
25	6	0	-0.613473	-0.294638	1.843527
26	6	0	0.631621	-0.420132	0.964287
27	1	0	1.460902	-0.037957	1.562081
28	1	0	0.529256	0.247311	0.100205
29	6	0	-0.438915	-2.374750	-0.264534
30	8	0	-0.571924	0.349577	2.876789
31	6	0	3.469025	-2.149606	-0.105678
32	6	0	3.804139	-0.761797	0.481675
33	6	0	3.604123	0.443094	-0.359338
34	1	0	3.428075	0.297592	-1.419730
35	6	0	3.550438	1.661613	0.194818
36	1	0	3.697573	1.741322	1.271039
37	8	0	4.210891	-0.699644	1.638700
38	6	0	3.261613	2.974858	-0.501290

39	6	0	2.953313	2.833620	-1.992991
40	1	0	2.091645	2.177475	-2.152684
41	1	0	3.816312	2.409812	-2.517577
42	1	0	2.731480	3.811716	-2.420598
43	6	0	4.434617	3.935788	-0.241755
44	1	0	4.594126	4.062256	0.834130
45	1	0	4.236143	4.911075	-0.687898
46	1	0	5.350552	3.521685	-0.677128
47	8	0	2.060815	3.412840	0.230387
48	6	0	1.476314	4.616453	-0.016795
49	8	0	1.871223	5.419854	-0.833736
50	6	0	0.254044	4.795886	0.853142
51	1	0	0.044081	5.862300	0.953299
52	1	0	0.398247	4.339149	1.835345
53	1	0	-0.617547	4.321354	0.385406
54	6	0	-1.693468	-2.329548	0.654023
55	1	0	-1.481830	-3.038244	1.461110
56	6	0	-2.845161	-1.180710	2.596638
57	1	0	-2.449778	-1.992820	3.216738
58	1	0	-3.859786	-1.451533	2.288591
59	1	0	-2.889942	-0.291600	3.227255
60	6	0	0.834234	-1.887212	0.538842
61	6	0	1.958874	-2.218930	-0.499559
62	1	0	1.841865	-1.528168	-1.340172
63	6	0	1.537427	-3.621766	-1.029647
64	1	0	2.136449	-4.396187	-0.535686
65	6	0	0.025695	-3.793872	-0.679488
66	1	0	-0.104122	-4.513998	0.135869
67	1	0	-0.520792	-4.181581	-1.546523
68	6	0	1.059335	-2.680856	1.856669
69	1	0	1.256548	-3.742003	1.698591
70	1	0	0.210561	-2.590299	2.541967
71	1	0	1.926179	-2.272068	2.379593
72	6	0	-0.651963	-1.597044	-1.598379
73	1	0	0.033276	-1.943858	-2.374027
74	1	0	-0.522163	-0.515416	-1.511479
75	1	0	-1.664410	-1.770814	-1.973004
76	8	0	1.790559	-3.648269	-2.440868
77	1	0	1.637595	-4.554781	-2.750442
78	8	0	3.793164	-3.130749	0.863061
79	1	0	4.120521	-2.627159	1.636557
80	6	0	4.378163	-2.393175	-1.332607
81	1	0	5.425976	-2.275407	-1.036424
82	1	0	4.162772	-1.717299	-2.165181

83 1 0 4.232496 -3.413775 -1.690603

Excitation energies and oscillator strengths:

Excited State 1: Singlet-A 3.8341 eV 323.37 nm f=0.0008 <S**2>=0.000
141 ->148 -0.31499
142 ->148 -0.32647
143 ->148 0.39681
145 ->148 -0.22610
147 ->148 -0.24924
This state for optimization and/or second-order correction.
Total Energy, E(TD-HF/TD-KS) = -1809.48493809
Copying the excited state density for this state as the 1-particle RhoCI density.

Excited State 2: Singlet-A 4.0056 eV 309.53 nm f=0.0067 <S**2>=0.000
141 ->148 -0.11283
143 ->148 0.17093
145 ->148 -0.11358
147 ->148 0.65385

Excited State 3: Singlet-A 4.1534 eV 298.51 nm f=0.0007 <S**2>=0.000
146 ->149 -0.17605
147 ->149 0.66798

Excited State 4: Singlet-A 4.3864 eV 282.66 nm f=0.0178 <S**2>=0.000
142 ->148 0.34935
143 ->148 0.21215
144 ->148 0.50875
146 ->148 0.25140

Excited State 5: Singlet-A 4.5277 eV 273.84 nm f=0.0028 <S**2>=0.000
143 ->148 -0.10712
144 ->148 -0.20383
146 ->148 0.65470

Excited State 6: Singlet-A 4.5844 eV 270.45 nm f=0.0205 <S**2>=0.000
142 ->148 -0.32206
144 ->148 0.29100
145 ->148 0.54986

Excited State 7: Singlet-A 4.8010 eV 258.25 nm f=0.0000 <S**2>=0.000
141 ->148 -0.23009
142 ->148 0.35990
143 ->148 0.31822

144 ->148 -0.30413
145 ->148 0.34946

Excited State 8: Singlet-A 4.8717 eV 254.50 nm f=0.0006 <S**2>=0.000
141 ->148 0.56031
142 ->148 -0.12217
143 ->148 0.38691
144 ->148 -0.12075

Excited State 9: Singlet-A 5.3404 eV 232.16 nm f=0.0634 <S**2>=0.000
137 ->148 0.48788
138 ->148 -0.18796
140 ->148 0.46281

Excited State 10: Singlet-A 5.4843 eV 226.07 nm f=0.0021 <S**2>=0.000
146 ->149 0.67612
147 ->149 0.19088

Excited State 11: Singlet-A 5.5499 eV 223.40 nm f=0.0039 <S**2>=0.000
137 ->148 0.14794
138 ->148 0.67303
140 ->148 0.11302

Excited State 12: Singlet-A 5.5798 eV 222.20 nm f=0.0039 <S**2>=0.000
142 ->151 0.15976
143 ->151 0.40428
143 ->152 0.15415
144 ->151 -0.27667
144 ->152 -0.11522
145 ->151 0.36370
145 ->152 0.12091
147 ->151 0.10005

Excited State 13: Singlet-A 5.6390 eV 219.87 nm f=0.0016 <S**2>=0.000
139 ->148 0.69940

Excited State 14: Singlet-A 5.6861 eV 218.05 nm f=0.0026 <S**2>=0.000
142 ->151 0.12625
142 ->152 -0.24711
144 ->151 -0.16938
144 ->152 0.32499
145 ->151 -0.21569
145 ->152 0.47234

Excited State 15: Singlet-A 5.7244 eV 216.59 nm f=0.2601 <S**2>=0.000
137 ->148 -0.47643
140 ->148 0.48522

Excited State 16: Singlet-A 5.8346 eV 212.50 nm f=0.0092 <S**2>=0.000
131 ->148 0.13636
132 ->148 -0.10559
134 ->148 0.25335
135 ->148 0.17137
136 ->148 0.58123

Excited State 17: Singlet-A 5.8412 eV 212.26 nm f=0.0052 <S**2>=0.000
146 ->150 -0.11752
147 ->150 0.66432

Excited State 18: Singlet-A 5.8722 eV 211.14 nm f=0.0051 <S**2>=0.000
143 ->149 0.20930
144 ->149 -0.43113
145 ->149 0.48490
147 ->150 -0.11221

Excited State 19: Singlet-A 5.9183 eV 209.49 nm f=0.0026 <S**2>=0.000
142 ->149 0.44219
143 ->149 0.22181
144 ->149 0.42825
145 ->149 0.19501
147 ->150 -0.10139

Excited State 20: Singlet-A 5.9538 eV 208.24 nm f=0.0053 <S**2>=0.000
142 ->149 -0.20559
143 ->149 0.61931
145 ->149 -0.23934

Excited State 21: Singlet-A 5.9829 eV 207.23 nm f=0.0049 <S**2>=0.000
129 ->148 0.11466
132 ->148 -0.13343
133 ->148 0.18118
134 ->148 0.26068
135 ->148 0.48432
136 ->148 -0.31862

Excited State 22: Singlet-A 6.0466 eV 205.05 nm f=0.0004 <S**2>=0.000
142 ->149 0.48102
144 ->149 -0.34271

145 ->149 -0.38078

Excited State 23: Singlet-A 6.1439 eV 201.80 nm f=0.0001 <S**2>=0.000
146 ->151 0.10701
147 ->151 0.67345
147 ->152 0.13778

Excited State 24: Singlet-A 6.1846 eV 200.47 nm f=0.0008 <S**2>=0.000
132 ->148 -0.13010
133 ->148 0.47147
134 ->148 0.25162
135 ->148 -0.41287

Excited State 25: Singlet-A 6.2094 eV 199.67 nm f=0.0014 <S**2>=0.000
141 ->149 0.69534

Excited State 26: Singlet-A 6.3057 eV 196.62 nm f=0.0036 <S**2>=0.000
129 ->148 -0.11180
132 ->148 0.27487
133 ->148 0.46764
134 ->148 -0.33396
135 ->148 0.20144
136 ->148 0.13241

Excited State 27: Singlet-A 6.3504 eV 195.24 nm f=0.0023 <S**2>=0.000
132 ->148 0.56900
134 ->148 0.35355

Excited State 28: Singlet-A 6.3809 eV 194.31 nm f=0.0042 <S**2>=0.000
146 ->151 0.65976
146 ->152 0.17771
147 ->151 -0.12594

Excited State 29: Singlet-A 6.4311 eV 192.79 nm f=0.0004 <S**2>=0.000
142 ->150 0.16288
143 ->150 0.39654
144 ->150 -0.33797
145 ->150 0.43354

Excited State 30: Singlet-A 6.4719 eV 191.57 nm f=0.0007 <S**2>=0.000
147 ->151 -0.12674
147 ->152 0.68511

Fig. S1 ^{1}H NMR spectrum of **1** ($CDCl_3$, 500 MHz)

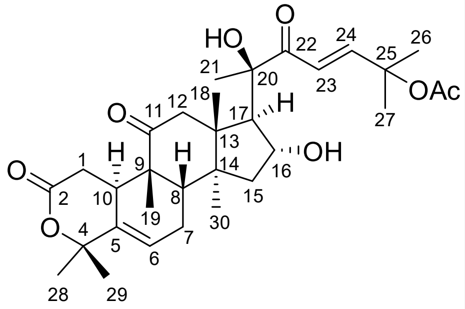

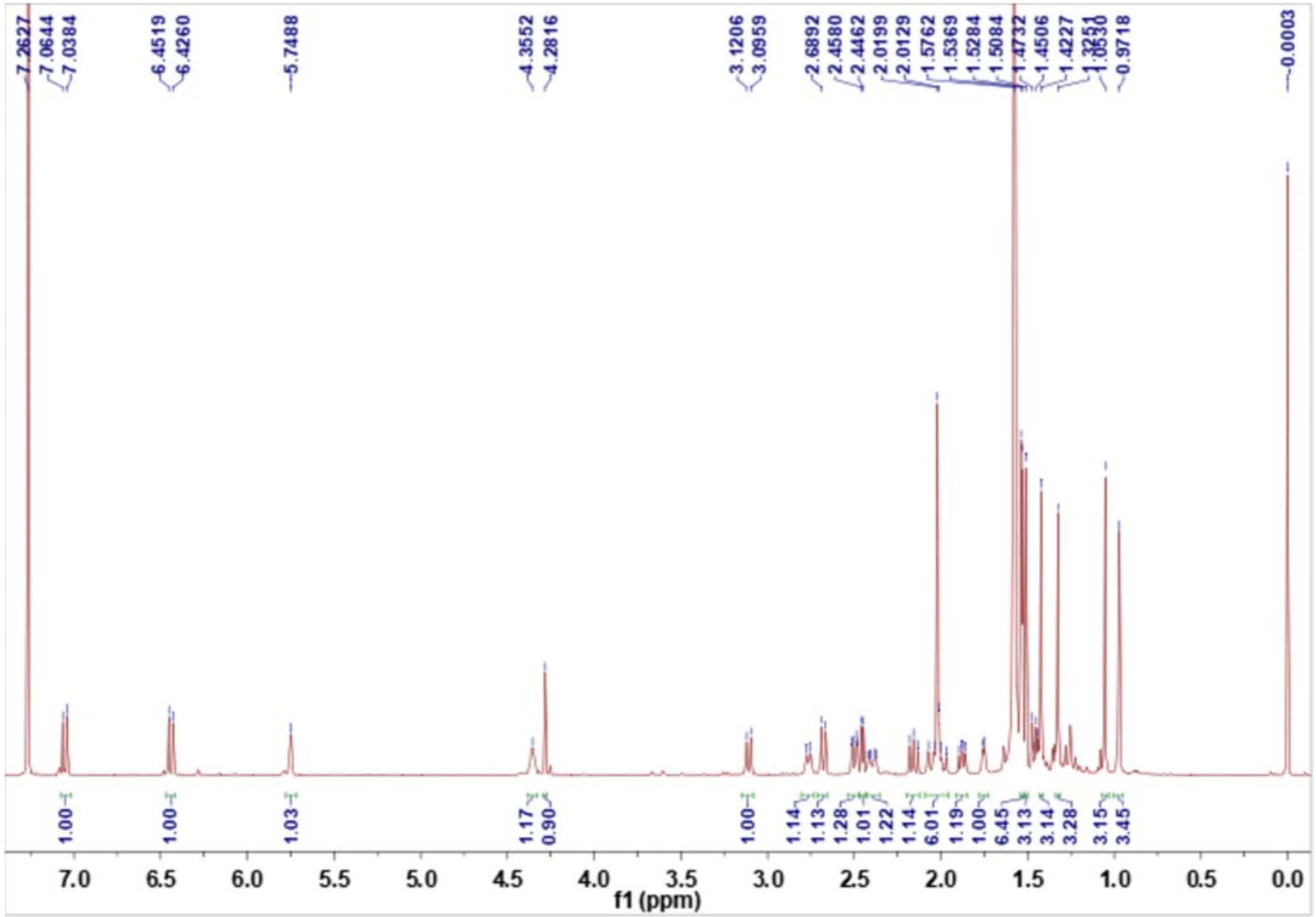

Fig. S2 ^{13}C NMR spectrum of **1** ($CDCl_3$, 126 MHz)

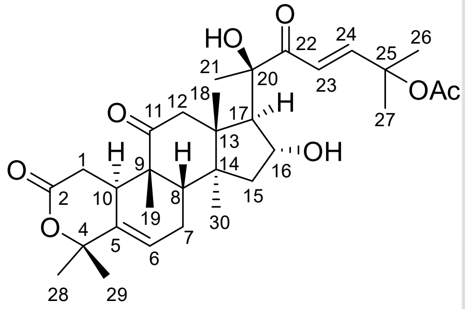

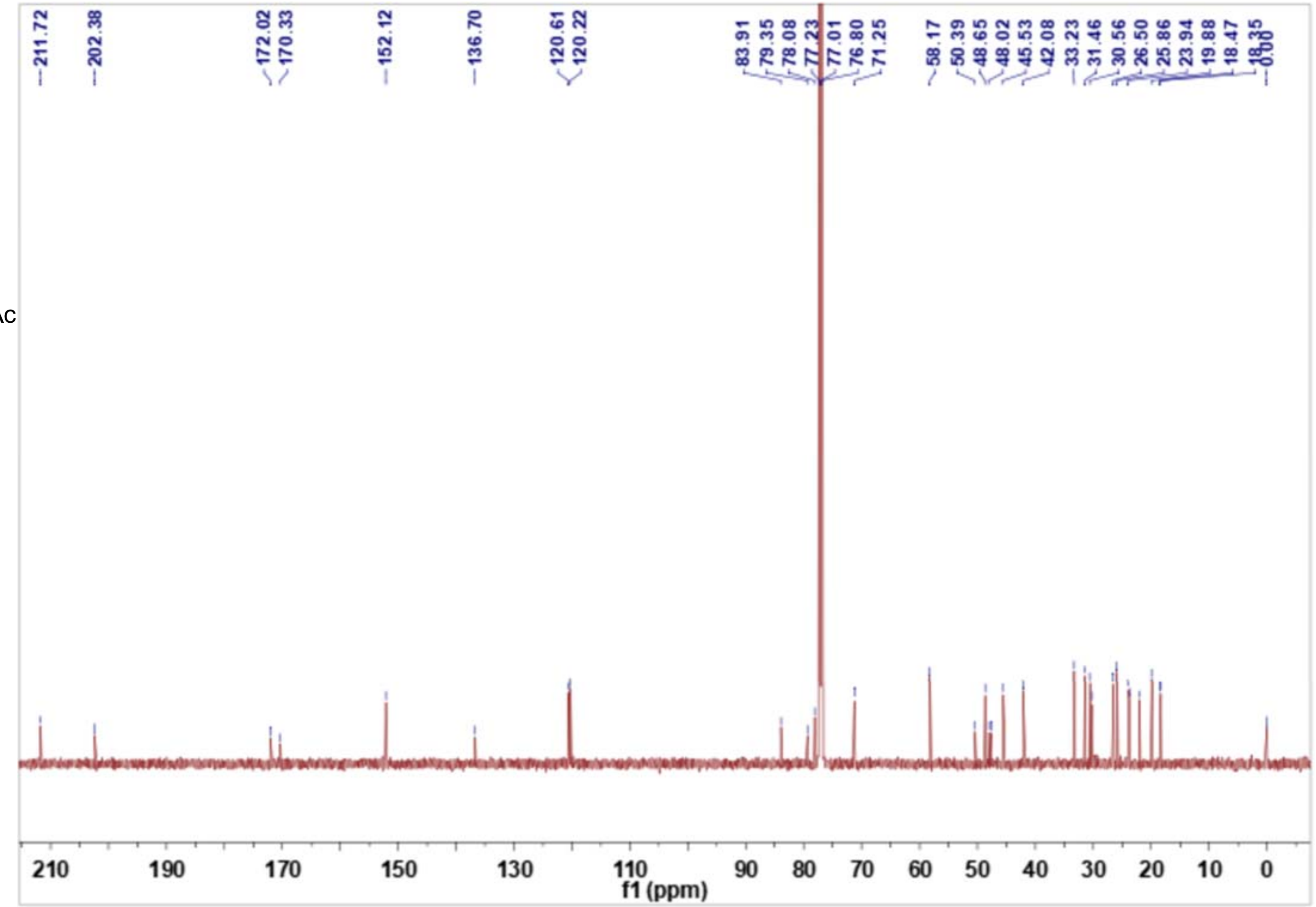

Fig. S3 HSQC spectrum of **1**

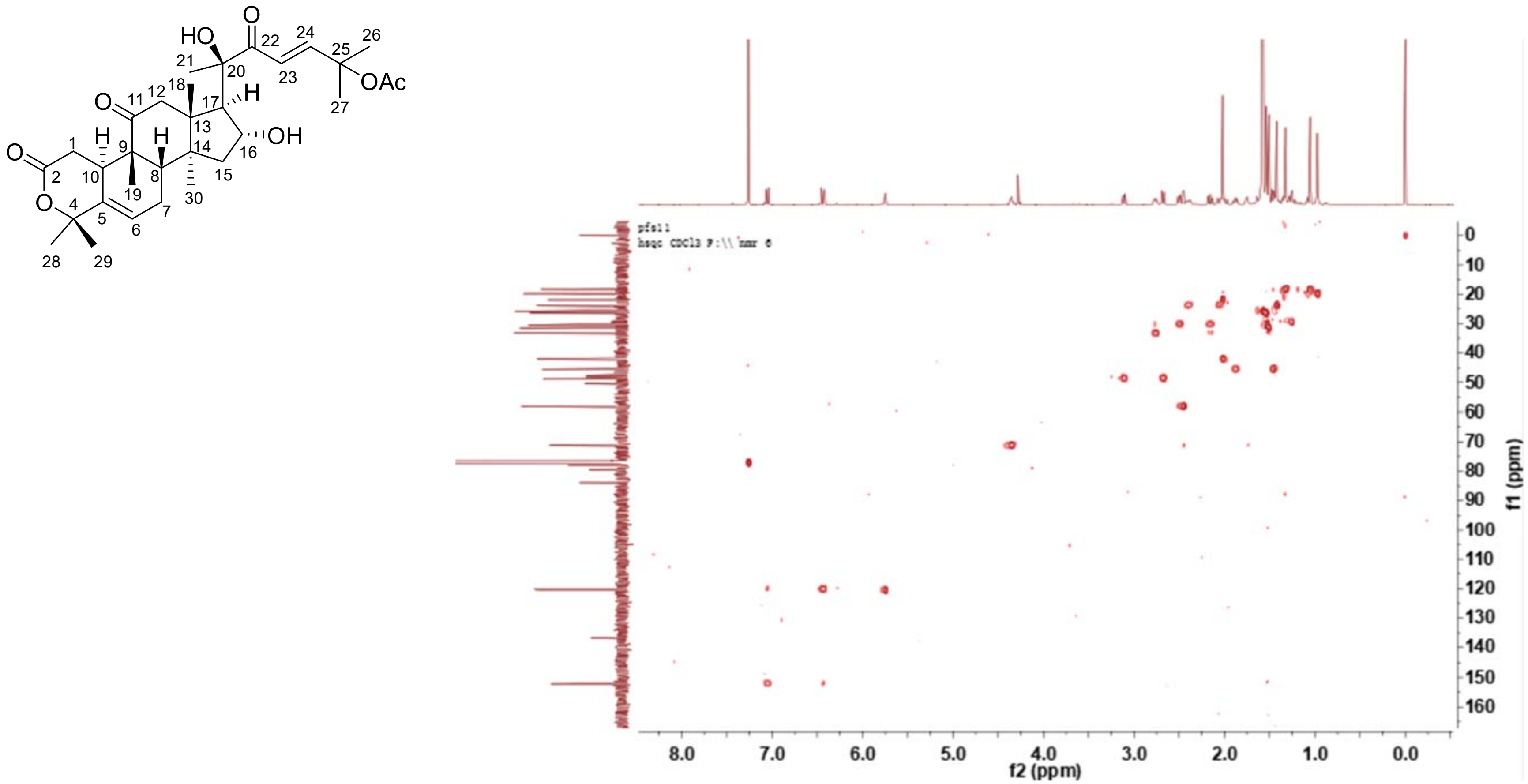

Fig. S4 ^1H–^1H COSY spectrum of **1**

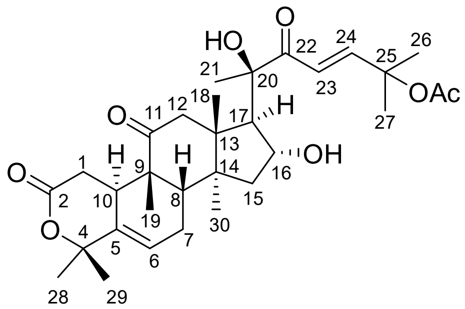

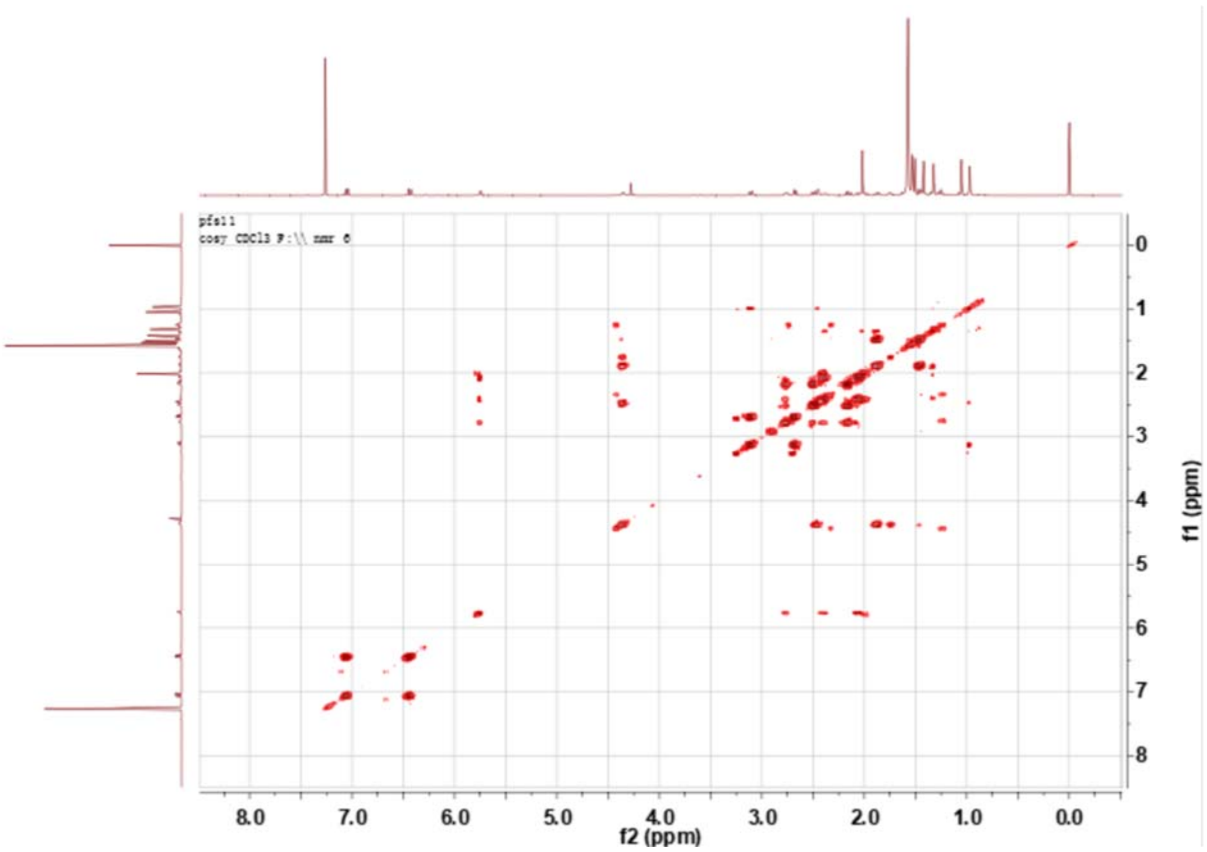

Fig. S5 HMBC spectrum of **1**

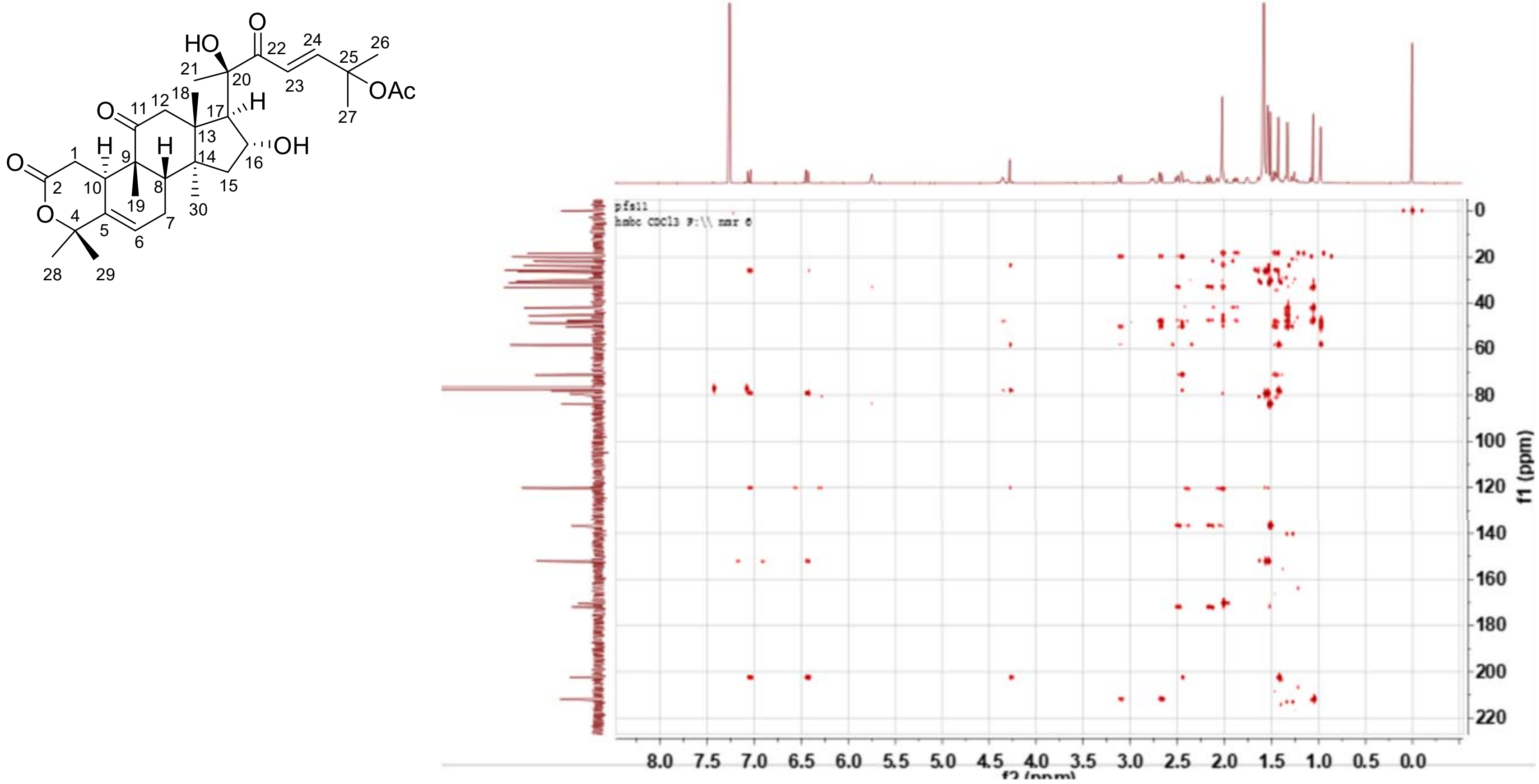

Fig. S6 ROESY spectrum of **1**

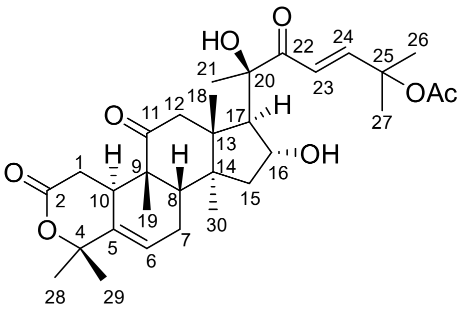

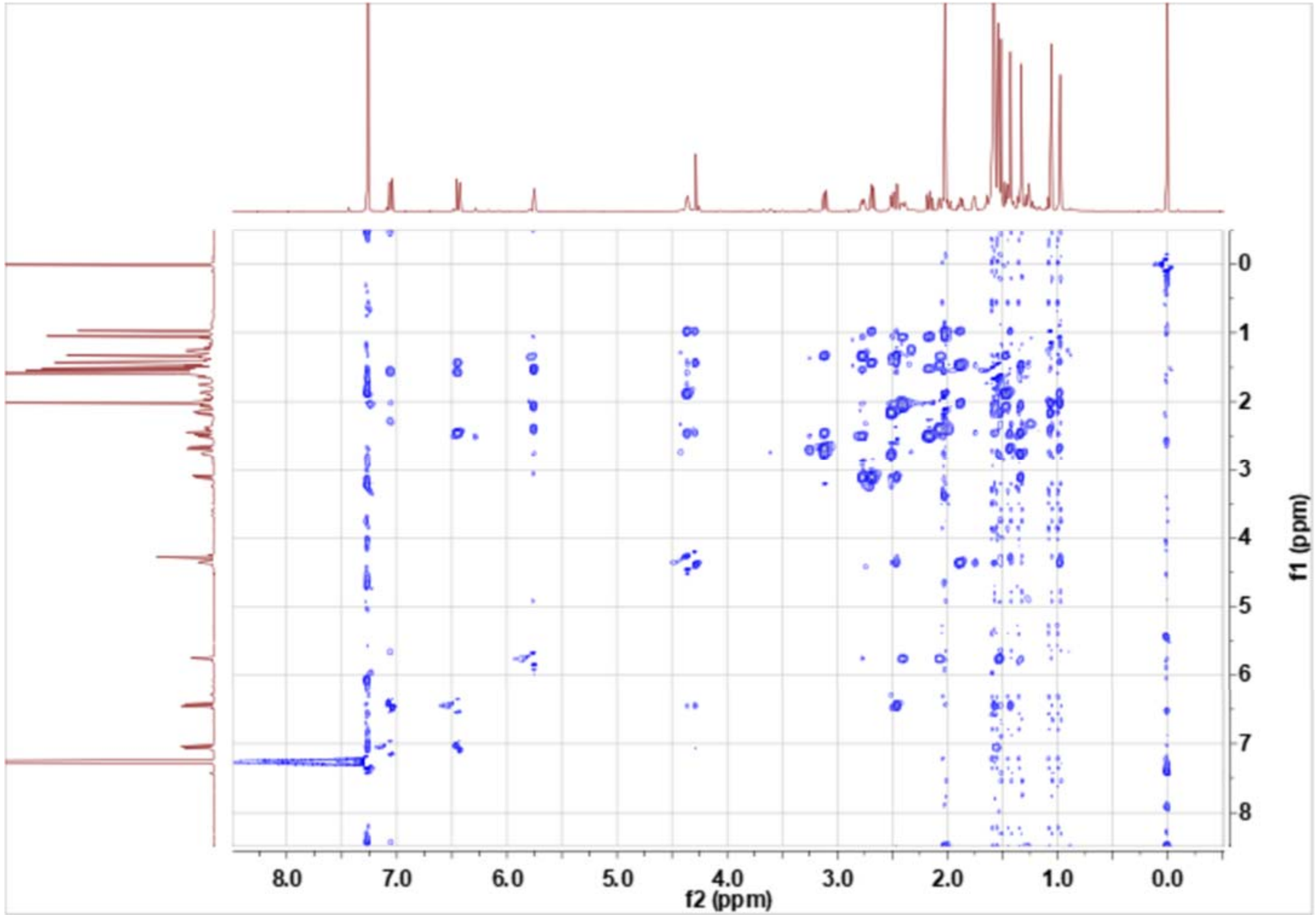

Fig. S7 HRESIMS spectrum of **1**

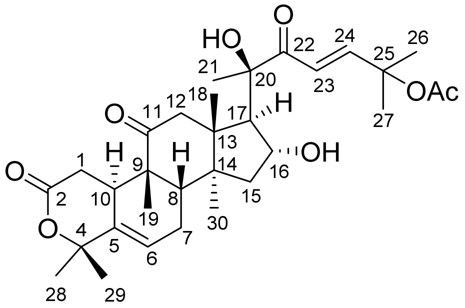

**Data Filename**	20201127ESIA2.d	**Sample Name**	pfs11
**Sample Type**	Sample	**Position**	
**Instrument Name**	Agilent G6230 TOF MS	**User Name**	KIB
**Acq Method**	ESI.m	**Acquired Time**	11/27/2020 10:25:07 AM
**IRM Calibration Status**	Success	**DA Method**	ESI.m
**Comment**	+		

Sample Group **Info.**

Acquisition SW Version 6200 series TOF/6500 series Q-TOF B.05.01 (B5125.2)

User Spectra

Fragmentor Voltage	Collision Energy	Ionization Mode
260	0	ESI

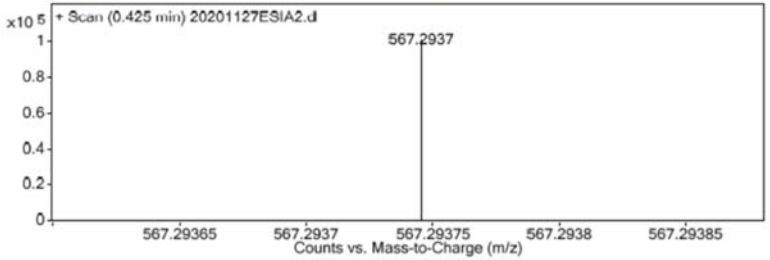

Peak List

m/z	z	Abund	Formula	Ion
226.1435	1	51763.8		
274.2739	1	82406.48		
384.3083	1	39532.35		
437.1937	1	130411.59		
438.1966	1	32968.9		
453.1673	1	36932.82		
507.2764	1	41213.43		
567.2937	1	100627.15	C31 H44 Na O8	M+
922.0098	1	409794.28		
923.0122	1	77236.63		

Formula Calculator Element Limits

Element	Min	Max
C	0	200
H	0	400
O	0	10
Na	1	1

Formula Calculator Results

Formula	CalculatedMass	Mz	Diff.(mDa)	Diff. (ppm)	DBE
C31 H44 Na O8	567.2934	567.2937	-0.3	0.5	9.5

--- End Of Report ---

Fig. S8 ECD spectrum of **1**

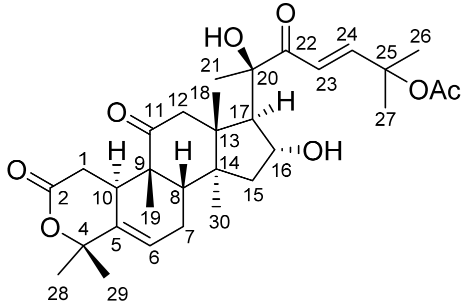

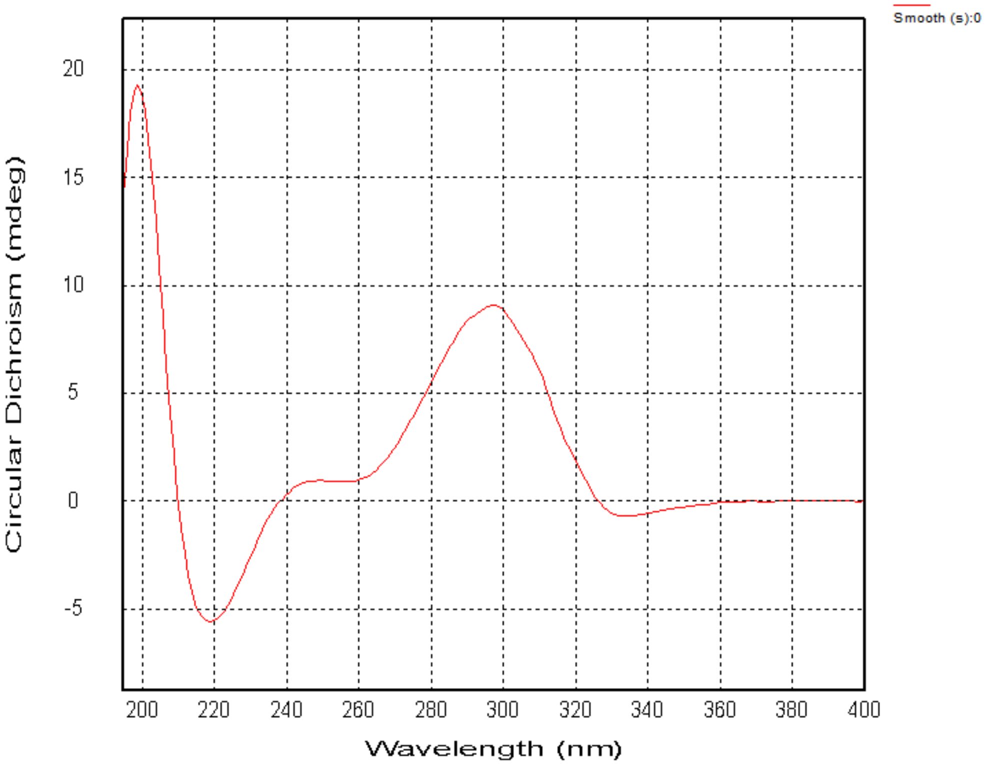

File: PFS11-1mm (195-400 nm) 20111703.dsx
ProBinaryX
Attributes :
- Time Stamp :Tue Nov 17 16:01:30 2020

- File ID : {880BFC30-F196-46a8-8F30-32EBAC41609F}
- Is CFR Compliant : false
- Original data has not been modified.

Remarks:
- User: CD
- Date: 2020/11/17
- Instrument: 0547
- DetectorType: LAAPD
- DichOS Calibration Correction Curve: 0547/2
- HV (CDDC channel): 0 v
- Time per point: 1 s
- Description: Sample 1
- Concentration: 0.5600mg/mL MeOH
- Pathlength: 1 mm
- Temperature: 20℃

Settings:
- Time-per-point: 1s (25us x 40000)
- SE
- Wavelength: 195nm - 400nm
